# Efficacy of traditional Chinese medicine combined with chemotherapy for gastric cancer and precancerous gastric lesions: a systematic review and meta-analysis

**DOI:** 10.3389/fonc.2026.1781660

**Published:** 2026-03-09

**Authors:** Xiao Liang, Liu Yang, Shuaiqi Yuan, Yueshan Zhang, Yuling Zheng

**Affiliations:** 1 Henan University of Chinese Medicine First Affiliated Hospital, Zhengzhou, Henan, China; 2 Anyang Tumor Hospital, The Affiliated Anyang Tumor Hospital of Henan University of Science and Technology, Anyang, Henan, China

**Keywords:** chemotherapy, gastric cancer, precancerous lesions, Quality of Life, TCM, traditional Chinese medicine, tumor response rate

## Abstract

**Background:**

Despite the advancements in therapeutic regimens, gastric cancer is still among the most concerning cancer-related public health problems. In previous literature, Traditional Chinese Medicine (TCM) proved to be an effective treatment strategy for reducing the side effects of chemotherapy and improving the quality of life among patients with gastric cancer or precancerous gastric lesions.

**Objectives:**

To analyze the efficacy of TCM combined with chemotherapy in individuals with gastric cancer and precancerous gastric lesions.

**Methods:**

PubMed, Cochrane Library, and Embase electronic databases were utilized to perform predefined search criteria for the identification and selection of studies based on eligibility criteria. The assessed outcomes include complete response rates (CRR), disease control rate (DCR), symptom scores, and quality of life (QoL), while the secondary outcomes include immune function, tumor markers, clinical symptom scores, and gastrointestinal adverse events. Random effect models were used to perform statistical tests (SMD: standardized mean difference, OR: odds ratio) with 95% confidence intervals and heterogeneity through I^2^ statistics. Publication bias was evaluated using funnel plots and tests like Egger’s regression or Begg’s correlation.

**Results:**

A total five studies reporting diverse outcomes were included in this meta-analysis. The SMD analysis for primary outcomes reported 0.35 [-0.66; 1.35] with considerable heterogeneity (I^2^ = 89.7%) while the CRR indicated remarkable improvement for combined therapy with OR of 4.20 [1.20; 14.71] and no heterogeneity. SMD analysis for QoL outcomes was also considerably high at 0.82 [-0.07; 1.81] with I^2^ = 80.0%. TCM combination group showed a significant difference in event rate with OR of 0.42 [0.32; 0.54], while the detailed analysis for symptom score of pooled -0.35 SMD [-0.76; 0.77] was obtained for fatigue, pain, acid reflux, nausea &vomiting, diarrhea. The publication bias assessment revealed significant asymmetry.

**Conclusion:**

The incorporation of TCM combined with chemotherapy for gastric cancer proved to be a safe and more effective treatment as the drug side effects were completely eradicated. The combination therapy has shown overall effectiveness with primary and secondary patient-reported outcomes, improving quality of life in gastric cancer individuals.

## 1 Introduction

Gastric cancer is the fifth most common cancer worldwide and the fifth most frequent cause of death, there are 968350 new cases and 659853 deaths in 2022, according to the GLOBOCAN 2022 database. Countries in East Asia, such as China, Japan, and Korea, carry the heaviest burden with high incidence rates that are markedly higher than in the West (1, 2). The situation, even though it is a little better, remains bad. The investment in health care and human lives lost currently, and the vast difference between the survival rate for advanced gastric cancer of about 20-30% after five years indicate very clearly the need for new, more potent treatment strategies (3–6).

Surgery, chemotherapy, and radiation therapy (in selected cases) are the cornerstones of the multimodal approach that constitutes the standard treatment for gastric cancer (7). A common chemotherapy regimen consists of doublets or triplets that include platinum-based agents (cisplatin or oxaliplatin) plus fluoropyrimidines (5-fluorouracil or capecitabine) and occasionally taxanes or anthracyclines (8, 9). Platinum-based therapeutic agents, such as cisplatin and oxaliplatin, are mostly preferred for the treatment of gastric cancer due to their unique mechanism of action. These agents form DNA crosslinks that prevent cancer cell replication, particularly effective against rapidly dividing cancerous cells in the gastric. Other meta-based chemotherapeutics are also present but platinum-based agents exhibit significant clinical efficacy and safety profiles in gastric cancer individuals, supporting evidence from clinical trials and recommendations from NCCV or ESMO guidelines.

While certain regimens have shown only slight advancements in overall survival and also progression-free survival in some cases, those treatments are still linked with high rates of myelosuppression, gastrointestinal disorders, peripheral nerve damage, and overall negativity of life as the main toxicity factors along with (10, 11). The above-mentioned unpleasant experiences quite often force the oncologists to either reduce the dosages or halt the treatment altogether leaving the patients vulnerable to cancer and probably a little bit worse than before with respect to the quality of life (12, 13).

Traditional Chinese Medicine (TCM) has been practiced in East Asia for thousands of years and is an all-encompassing medical system that aims at the body’s restoration of balance and harmony (13). The scope of TCM includes the use of various methods such as herbal medications, acupuncture, moxibustion, dietary therapy, and mind-body exercises. In the field of oncology, TCM is getting more and more combined with the conventional Western medicine, especially in China and other Asian countries, with the twofold goal of enhancing antitumor efficacy and relieving treatment-related toxicity (14, 15). Traditional TCM combinations such as Banxia Xiexin Decoction, Weifuchun, and Sijunzi Tang, are very often prescribed together with modern Chinese patent medicines in the case of gastric cancer patients undergoing chemotherapy (16).

The TCM theory states that the development of cancer is due to the imbalance in the vital energy (Qi), blood, yin-yang, and organ systems (17). It is assumed that by working through various mechanisms, TCM herbal formulations will lead to immune modulation, apoptosis induction, angiogenesis inhibition, and enhancement of chemo-sensitivity (18, 19). Preclinical studies have revealed a large number of bioactive compounds in TCM herbs with possible anti-cancer properties like ginsenosides, flavonoids, polysaccharides, and alkaloids (20, 21). These compounds may work together to interfere with various signaling pathways associated with tumor growth, metastasis, and drug resistance (22, 23).

The Correa cascade of gastric carcinogenesis comprises several successive events; precancerous gastric lesions, such as chronic atrophic gastritis, intestinal metaplasia, and dysplasia, are among the most critical ones (24, 25). Intervention at the very beginning of the process not only offers the possibility of preventing but also of delaying the transition toward invasive cancer. The applicability of TCM has been explored more and more, especially in terms of its capability to reverse or stop the process of precancer development due to its anti-inflammatory, antioxidant and mucosal protective properties (26, 27).

The efficacy of TCM in conjunction with chemotherapy for the treatment of gastric cancer and precancerous conditions, even though it is already recognized by clinical practice and research is still considered to be not well characterized. Previous research reviews have been limited in scope due to the small number of patients, different outcome measures, and the poor quality of methodology that have been considered (28). Not only that, but also, it is still unknown which TCM formulations, dosing schedules, and patient types would be the most responsive to the treatment.

This systematic review and meta-analysis was designed to be a comprehensive evaluation of the existing evidence on the efficacy and safety of TCM plus chemotherapy *vs*. chemotherapy alone or standard treatment in gastric cancer and precancerous gastric lesions patients. By retrieving the data from randomized controlled trials (RCTs), the goal of this study is to provide solid evidence that will not only guide clinical practice but also reveal the directions for further research.

## 2 Methods

### 2.1 Design & protocol

This systematic review and meta-analysis were carried out by following the directions of Preferred Reporting Items for Systematic Review and Meta-Analysis (PRISMA) (29). These guidelines were required for conducting an effective search protocol necessary for the identification, screening, and selection of the target studies reporting the treatment for gastric cancer through TCM combined with chemotherapy and compared with controlled or placebo group. The need for ethical review was eliminated because the selected studies were already published.

### 2.2 PICO framework

- Population (P): Individuals (≥ 18 years) diagnosed with histologically confirmed gastric or cardia cancer of any stage and precancerous gastric lesions (chronic atrophic gastritis, intestinal metaplasia, or dysplasia). Gastric cancer patients of any gender, ethnicity, and nationality can be included.
- Intervention (I): Traditional Chinese Medicine (TCM) formulations of any type or combined with standard chemotherapy included either classical herbal decoctions (Banxia Xiexin Decoctin, Weifuchun, Sijunzi Tang) or Chinese patent medicines like capsules/tablets combined with Western chemotherapy.
- Comparator (C): Standard, placebo, or control treatment including chemotherapy alone, chemotherapy combined with placebo, and other conventional therapies for gastric cancer individuals.
- Outcomes (O): Primary outcomes include tumor complete response rate (CRR), disease control rate (DCR), disease-free survival (DFS), progression-free survival (PFS), overall survival (OS), and histologically improved outcomes for precancerous lesions. Secondary outcomes include immune function indices, tumor markers, clinical symptom scores, quality of life (QoL), and gastrointestinal adverse events.
- Study Design: Randomized Controlled Trials (RCTs) or Controlled Clinical Trials (CCTs).

### 2.3 Search strategy

A systematic search was conducted across three major databases, PubMed, Cochrane CENTRAL, and Embase, resulting in the identification of eligible records. This search strategy was designed to find studies evaluating therapeutic efficacy of TCM combined with chemotherapy for gastric cancer and precancerous lesions of gastric cancer. The complete search strategy of the literature studies was presented in the Supplementary Methods section. The search strategy was designed by incorporating MeSH terms related to TCM (“Medicine, Chinese Traditional”, “Drugs, Chinese Herbal”, or “Complementary Therapies”), gastric cancer (“Stomach Neoplasms”, “Precancerous Conditions”, or “Gastritis, Atrophic”), and chemotherapy (“Drug Therapy” or “Antineoplastic Agents”). While other text keywords include herbal formula, herbal remedies, integrated medicine, Banxia Xiexin Decoctin, Shenqi Fuzheng, Ydanzi, Xiaoyao, Shiquan Dabu, and many other traditional medicine herbs utilized in treating gastric cancer or precancerous gastric lesions. Meanwhile, the cancer related (gastric cancer, precancerous lesions, intestinal metaplasia, or dysplasia) and therapy-related (5-fluorouracil, oxaliplatin, paclitaxel, or cisplatin) were also incorporated to search literature studies. The MeSH terms and keywords were integrated with Boolean operators (AND/OR/NOR/NOT) to define the search criteria for the identification of research studies reporting TCM combined with chemotherapy for gastric cancer and precancerous gastric lesions. By incorporating these literature search terms, the records were identified, screened, and assessed for eligibility, and the final valid studies were included in the meta-analysis.

### 2.4 Eligibility criteria

The inclusion criteria involved RCT or Quasi-controlled trials in which human individuals are diagnosed with gastric/cardia cancer or precancerous lesions receiving any of herbal TCM combined with chemotherapy, while the comparator group includes either chemotherapy alone or standard therapy. Studies reporting at least one of the predefined primary or secondary outcomes with sufficient data were included for further meta-analysis. Meanwhile, the exclusion criteria include non-randomized, observational studies, case reports, case series, review articles, systematic reviews, meta-analyses, study protocols, and retracted articles. Studies reporting mixed cancer populations or other than gastric cancer individuals receiving TCM alone without chemotherapy, unclear TCM composition, or given as pre-treatment, while there was no data on post-treatment reported, were excluded. Animal and *in vitro* studies were also excluded. Moreover, non-full-text articles, duplicate publications, retracted articles, studies with methodological problems, or studies with insufficient outcome data were removed. The search literature were exported to the Zotero library and assessed for eligibility for inclusion in systematic review and meta-analysis.

### 2.5 Data extraction process

Data extraction was performed independently by two reviewers through a standardized, pre-defined data extraction table as represented in tabular form. Any discrepancies were resolved either through discussion or by the involvement of third reviewer. The standardized data extraction form include information related to study characteristics (author, year of publication, country, design, setting, or duration), population characteristics (sample size, age, gender distribution, disease type, diagnosis criteria, or disease stage), intervention (TCM combination, dose, route of administration, and combination with other therapies), comparator, and clinical outcomes (CRR, DCR, survival rate, symptom, QoL, markers, and adverse events) among intervention and control groups. For outcomes reported in the included studies, the mean (standard deviation/SD) and event rate were extracted to further calculate effect size.

### 2.6 Risk of bias assessment

The methodological quality and risk of bias of the included RCT studies were assessed by two reviewers through the Cochrane Risk of Bias (RoB 2.0) tool (30), encompassing the five domains like randomization process (selection bias), allocation concealment or blinding (selection or performance bias), blinding of outcome assessment (detection bias), missing outcome data (attrition bias), and selective reporting bias (reporting bias). Each RoB domain was rated as low (green or +), high (red or -), or unclear risk of bias (yellow or)! based on criteria specified in the Cochrane Handbook. After assessing the risk of biasness of individual studies in each domain, an overall risk of biasness was summarized and evaluated the quality of the studies. The data extraction and risk of bias assessment was done by two reviewers independently and any disagreements among the reviewers were resolved through discussion and the involvement of third reviewer if necessary.

### 2.7 Statistical analysis

Meta-analyses were performed using RStudio statistical software (version 2025.05.0 Build 496) along with the meta and metafor packages. Standardized mean differences (SMD) with 95% confidence intervals (CI) were computed for continuous outcomes (symptom scores, quality of life measures, indices of immune function, and tumor markers). SMD was calculated using Hedges’ g formula which compensates for small sample sizes:

SMD = x̄1 - x̄2/Sp.

where pooled Sp represents the pooled SD, allowing comparison across studies using different measurement scales and following recommendations from the Cochrane Handbook and Cohen’s methodological framework (31). For studies reporting medians and ranges, we used the method adopted to estimate means and SDs. The random effects model was implemented using the DerSimmonian-Larid method and effect size were interpreted using Cohen’s guidelines (32, 33). On the other hand, for dichotomous outcomes (such as tumor response rates, adverse events, and histological improvement), odds ratios (OR) or risk ratios (RR) along with 95% CI were determined. The p-value of >0.05 or >0.001 was considered significant. Moreover, the publication bias was evaluated through Egger’s linear regression and Begg’s correlation test to assess funnel plot symmetry.

### 2.8 Heterogeneity assessment

This meta-analysis used random-effect models to assess the homogeneity of the combined trials or mixed study designs. Cochrane’s Q, a weighted sum of the squared differences between individual and pooled effects across studies, was used to quantify the degree of heterogeneity regarding the prognostic value of ctDNA mutation panels in predicting early recurrence and survival outcomes for early-stage breast cancer patients. Given that the chi-square test for heterogeneity is a low-power test, the alpha level was set at 0.10. The I-squared (I^2^) score was then used to gauge the degree of heterogeneity, and any score of 40% or more was investigated using sensitivity and subgroup analysis.

### 2.9 Subgroup and sensitivity analysis

Given the degree of heterogeneity, subgroup analysis was conducted based on outcomes reported in the included studies for symptoms to determine the impact of these factors on ctDNA detection. Sensitivity leave-one-out analysis was also performed, which explains the effect of omitting a single study on the pooled effect size and heterogeneity, and to evaluate the robustness of the results by testing their dependence on the study quality.

## 3 Results

### 3.1 Identification of eligible studies

**Figure 1:**
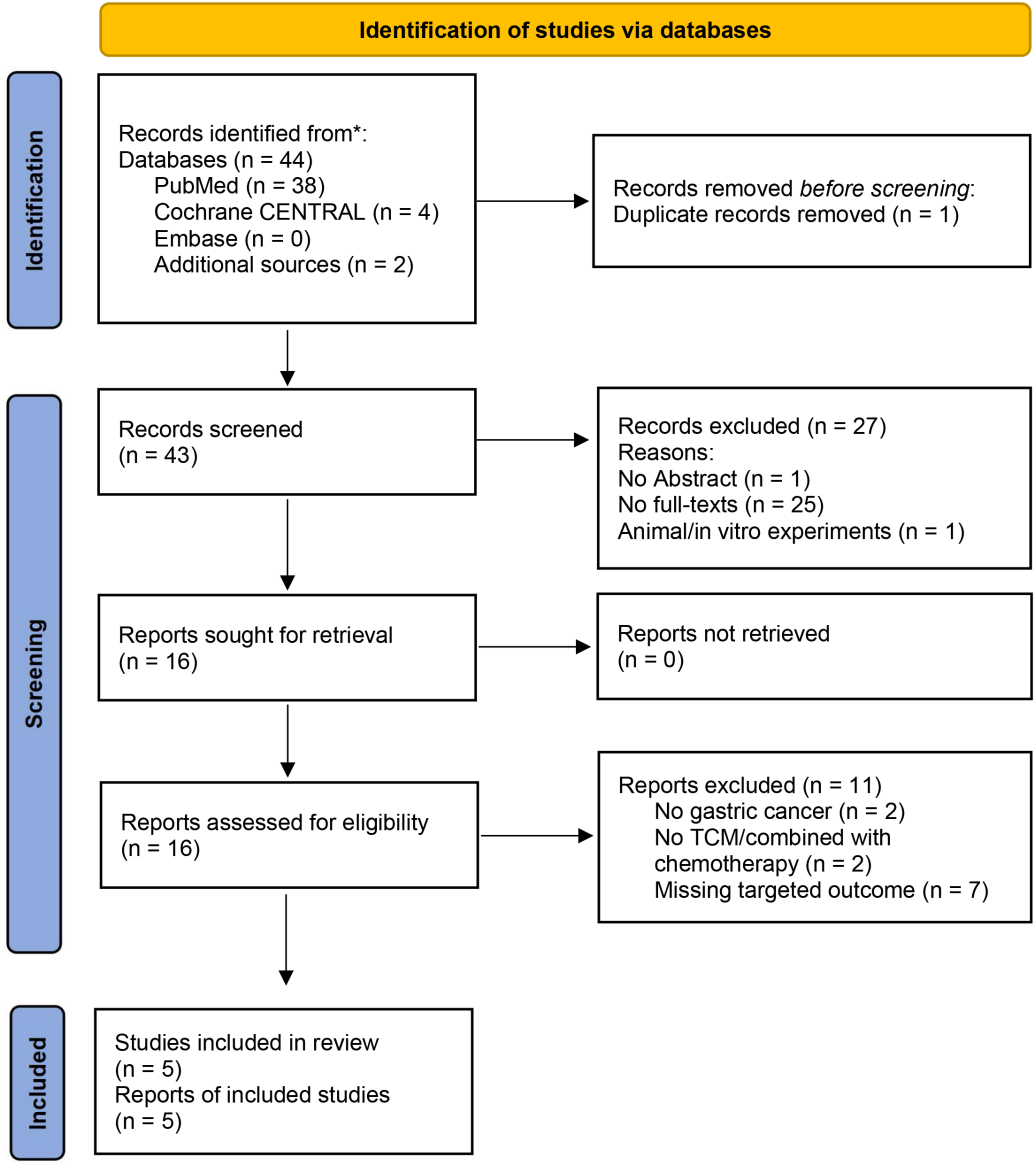
Search strategy and protocol defined for the identification, screening, selection, and inclusion of the studies based on Preferred Reporting Items for Systematic Reviews and Meta-Analysis (PRISMA) guidelines, where ‘n’ is the number of studies at each step of the search protocol.

A total of 44 research studies were identified by implementing a defined literature search under PRISMA guidelines (29) in major electronic databases PubMed (n = 38), Cochrane CENTRAL (n = 4), Embase (n = 0), and additional sources (n = 2) from which 5 publications were included in this meta-analysis. The systematic search strategy is illustrated in the PRISMA flow chart diagram, as shown in **Figure 1**. Out of 44 identified records, one duplicate records were removed, and 43 records were extensively screened. During screening, 27 records were excluded as these records were with no abstract (n = 1), no full-text articles (n = 25), and animal experiments (n = 1). Following screening, the 16 records were assessed for eligibility based on pre-defined selection criteria, and 11 records were excluded as some were not on gastric cancer (n = 2), some reported treatment other than TCM combined chemotherapy (n = 2), and missing targeted outcome (n = 7), while the remaining 5 studies (34–38) were included in this systematic review and meta-analysis.

### 3.2 Study characteristics

The finalized five studies included in this analysis represented a total of 821.4 participants, with an average of 403 in experimental groups and 418 in control groups, just like in **Table 1**. The studies were mainly conducted East Asian countries, with China being the most productive country and corresponds to the region’s extensive use of TCM in cancer care. Sample sizes varied from small pilot studies to large multi-center clinical trials. A wide variety of TCM were analyzed, including classical decoctions like Banxia Xiexin Decoction and Sijunzi Tang, and also various kinds of Chinese patent medicines. The chemotherapy regimens in both the intervention and the control group consisted of standard methods applied to gastric cancer. The studies differed in their participant characteristics, including patients with various cancer stages (from early-stage resectable disease to advanced metastatic cancer) and precancerous lesions at different stages of the Correa cascade. Treatment protocols varied from several weeks to many cycles of chemotherapy, reflecting the different practices in real-world clinical settings. Outcome measures reported across studies were heterogeneous, with varying assessment tools, timing of measurements, and definitions of response criteria. This methodological heterogeneity contributed to statistical heterogeneity in pooled analyses and necessitated careful interpretation of results.

**Table 1:** Characteristics of the included studies.

Study characteristics					Population characteristics						TCM characteristics				Control
Author/year	Country	Design	Setting	Duration	Sample size (I/C)	Age	Gender (M/F)	Disease type (cancer/lesion)	Diagnosis method/criteria	Disease stage	Composition	Dose	Route	Combination	Comparator
Kim et al., 2012	Germany	Randomized controlled pilot study	Hospital	2006-2008	32 (16/16)	53.75 (10.25)	2/3.	Gastric cancer	Eastern Cooperative Oncology Group (ECOG)	stage Ib, II	AbnobaVISCUM (endotoxin free plant extract)	20 mg	subcutaneous	5-fluorouracil prodrug 5-DFUR	placebo
Yuan et al., 2024	China	Controlled trial	Hospital	2022-2023	62 (29/33)	67.9 (11.3)	18/11	Gastric cancer		stage III, IV	Fuzheng Qingdu Decoction	one dose a day which was administered 2 times	oral	SOX chemotherapy	chemotherapy
Aoyama et al., 2014	Japan	Double-blind, placebo-controlled, randomized phase II study	Hospital		45/46	68 (36-84)	28/17	Gastric cancer with moderate-to-severe oral mucositis	CTCAE v4.0 grading	CTCAE v4.0 grade ≥1	TJ-14 (hangeshashinto)	2.5g three times per day	oral	chemotherapy	placebo
Lissoni et al., 2009	Milan	Randomized study	Hospital		240 (119/121)	65 (58-79)	65/54	Metastatic solid tumor	histological diagnosis		Aloe arborescens	10 ml Aloe thrice/daily	oral	chemotherapy (5-FU)	chemotherapy
Zhu et al., 2006	China	Randomized	Hospital		40 (20/20)			Late gastric antrum			Fuzheng Kang’ai Granules 48 hours after the first super-selective left gastric artery chemotherapy with high-dose drugs		oral	chemotherapy with high-dose drugs (EAP regime: VP(16) 100 mg/m(2) + epirubicin 60 mg/m(2) + carboplatin 200 mg/m(2))	Intra-arterial chemo alone

### 3.3 Quality or risk of bias assessment

**Figure 2:**
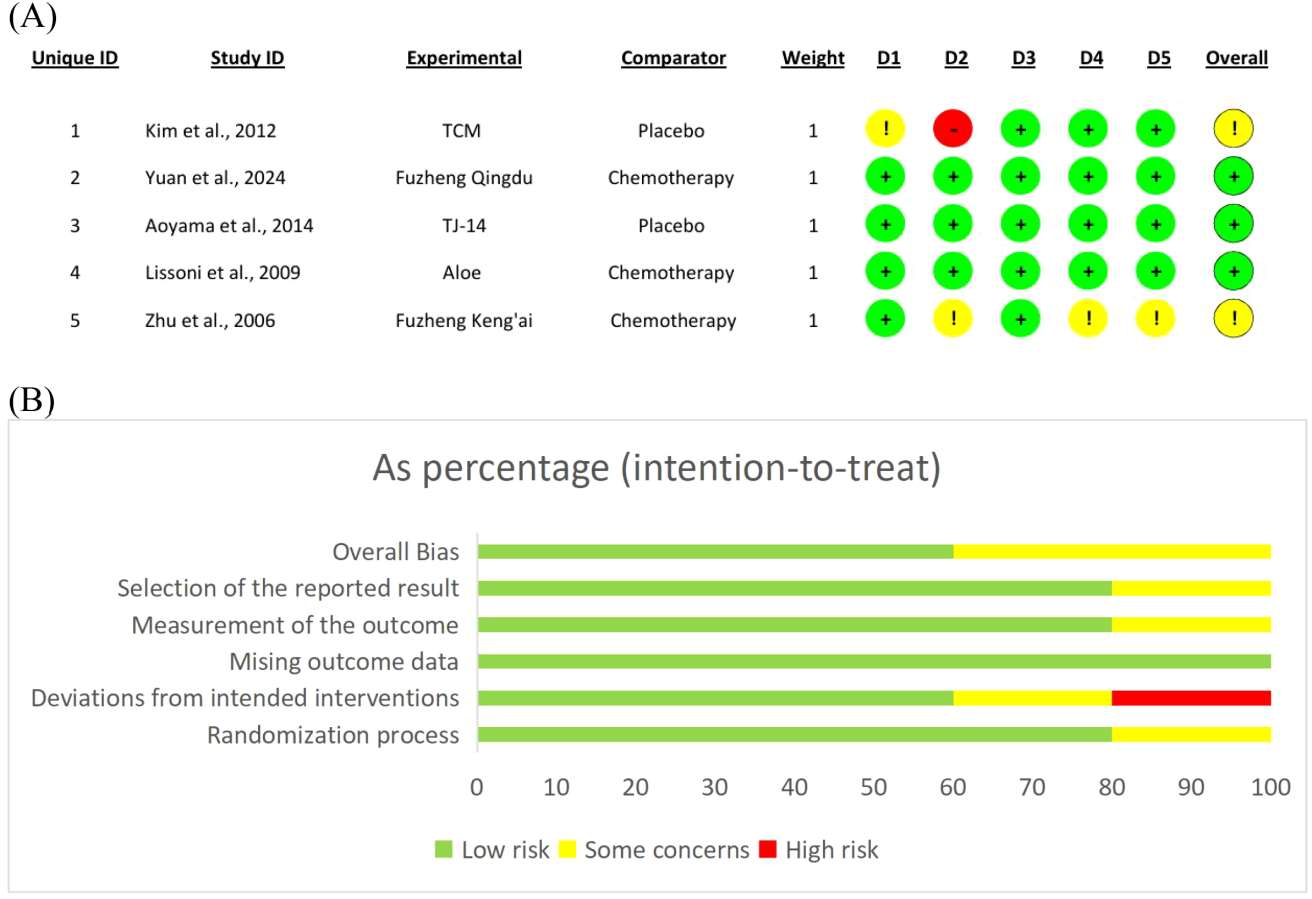
Risk of bias assessment for **(A)** individual studies and **(B)** summary of the included studies.

The assessment of risk of bias showed the presence of different methodological qualities in the studies that were included (**Figure 2**). Randomization was claimed in all studies, however, the adequacy of the random sequence generation was often unclear or poorly described. A number of the studies did not provide information about how they concealed the allocation of participants which is a major issue when it comes to the possibility of biases in the selection of participants. Usually, there were no measures taken to blind the participants and the staff which was probably due to the difficulty in blinding herbal treatments that have clear and pronounced tastes and smells and hence, they are easily detectable. Correspondingly, the blinding of the evaluators of the outcomes was not frequently mentioned either. The dropout rates were mostly in an acceptable range where the majority of the investigated cases were supplemented by full follow-up or application of proper techniques for dealing with the missing data, introducing potential attrition bias that may affect outcome reliability. Due to the lack of protocols published for the majority of the studies, the assessment of selective reporting bias was difficult. To sum up, the studies that were included gave useful information but the inconsistencies and unclear reporting in several risk of bias domains lead to the call for caution in the interpretation of findings.

### 3.4 Forest plot analysis for primary outcomes

**Figure 3:**
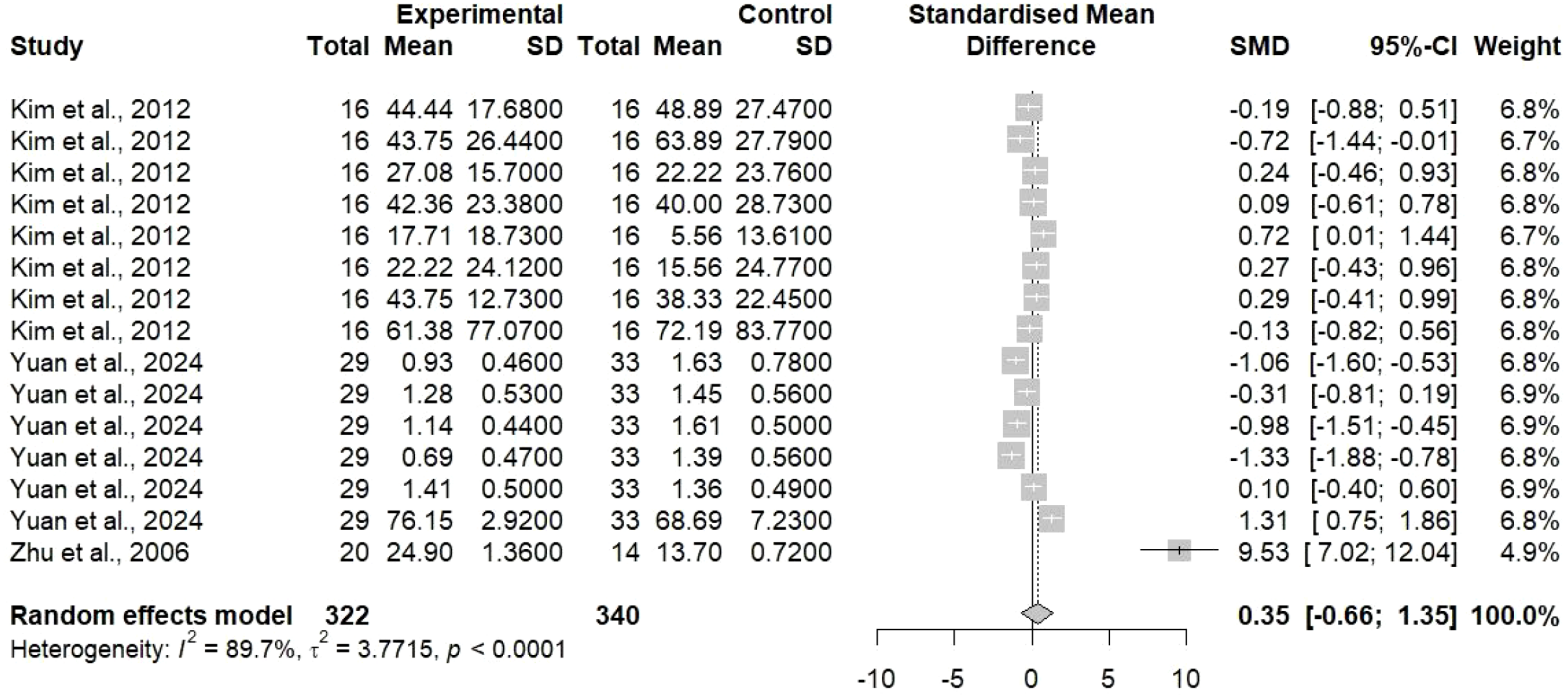
Standardized mean difference (SMD) analysis of outcomes reported in the included studies illustrated as an SMD forest plot with 95% confidence intervals (CI) using a random effects model. Pooled SMD with 95% CI was 0.35 [-0.66; 1.35] with heterogeneity of I^2^ = 89.7%. Where each square represents a single study, the central dotted line represents the line of no effect, and the diamond represents the overall effect size.

The random-effects model indicated that the pooled analysis of primary outcomes from the included studies resulted in a SMD of 0.35 (95% CI: -0.66 to 1.35), as presented in **Figure 3**. The pooled estimate was not statistically significant (the confidence interval crosses zero), thus the overall effect of TCM together with chemotherapy on primary outcomes was not clearly better than that of chemotherapy alone when all studies were considered.

Furthermore, a remarkable heterogeneity was noticeable (I² = 89.7%), which means that there was a huge difference in the effect sizes that were reported for outcomes by the different studies. This heterogeneity means that the effect of the treatment might differ based on the patient characteristics, TCM formulation used, chemotherapy regimen, the measure of outcomes or other factors related to the study. The wide confidence interval is a result of both the heterogeneity and the uncertainty in the pooled estimate. The forest plot showing the individual study results reveals strong difference, as some studies support TCM combination (positive SMD) and others report no gain or even adverse effects. One study (Zhu et al., 2006) reported an extremely large positive effect (SMD = 9.53), which might be an outlier that greatly affects the pooled estimate and adds to the heterogeneity.

**Figure 4:**
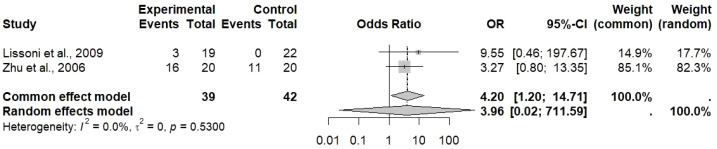
Odds ratio (OR) analysis with 95% CI for complete response rate reported within the included studies, where the observed effect size was 4.20 [1.20; 14.71] for the common effect model and 3.96 [0.02; 711.59] for the random effect model with heterogeneity of I^2^ = 0.0%.

Analysis of the CRR in **Figure 4** has shown more favorable results. The common effect model gave an odds ratio of 4.20 [95% CI: 1.20 to 14.71], signifying that there is a statistically significant fourfold increase in the likelihood of complete response with TCM combined chemotherapy as compared to chemotherapy alone. The random effect model produced a broader confidence interval with an OR of 3.96 [95% CI: 0.02 to 711.59] because of the small number of studies, requiring further validation by the inclusion of more studies reporting CRR for gastric cancer treatment. The heterogeneity for CRR amounted to 0% (I² = 0.0%), which is an important finding as it implies uniformity in the effects of treatment across the studies that were included for this outcome. Hence, this outcome gives stronger proof that TCM combination treatment can increase the rates of complete tumor response; however, the conclusion still cannot be definitive since only few studies were available.

### 3.5 Forest plot analysis for QoL and event rate

**Figure 5:**
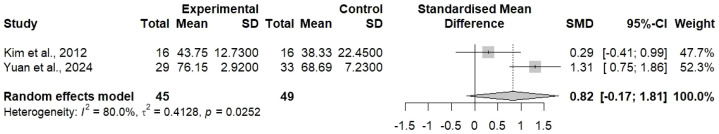
Pooled quality of life (QoL) outcome assessed for SMD analysis with 95% CI among the included studies using a random effects model. Overall SMD of 0.82 with 95% CI [-0.17; 1.81] was observed with a heterogeneity of I^2^ = 80.0%.

QoL) was one of the primary outcomes in the various studies and was assessed with the help of different instruments. The pooled SMD for QoL was found to be 0.82 [95% CI: -0.17 to 1.81] with a random-effects model which means that the TCM combination therapy might have a very large positive effect (**Figure 5**). It is inferred from the positive SMD that the patients taking TCM along with chemotherapy are likely to have a better QoL as compared to the ones receiving only chemotherapy; yet, the confidence interval includes zero, indicating that the result is not statistically significant at the p < 0.05 level. The QoL outcome showed a high level of heterogeneity (I² = 80.0%), which was due to different QoL assessment methods, different study populations, and different times of assessment and interventions across studies. The broad confidence interval also implies a lot of uncertainty regarding the benefit of QoL, and the borderline significance shows that there is a trend of improvement, but the evidence is still not enough for the final conclusion. The clinical significance of an SMD of 0.82, if confirmed, would be quite considerable, as it would be regarded as a large effect size by the standard of conventions. This level of improvement would most probably be perceived by the patients as clinically meaningful and would potentially be a reflection of the lower symptom burden, better functional capacity, and improved psychological well-being.

**Figure 6:**
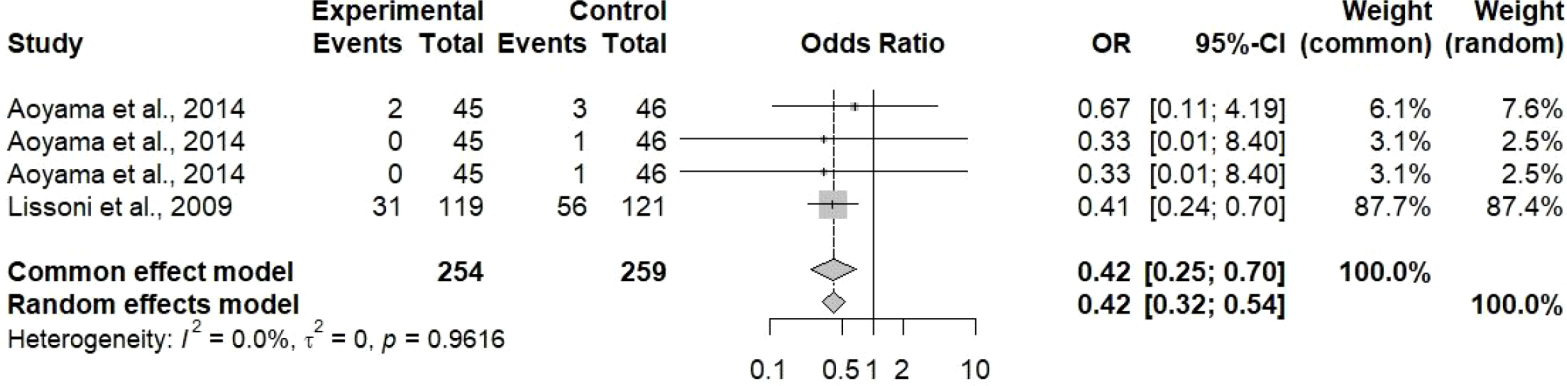
The pooled event rate for outcomes reported in the included studies was evaluated by illustrating an OR forest plot with 95% CI. The pooled OR of 0.42 [0.25; 0.70] for the common effect model and 0.42 [0.32; 0.54] was observed for outcomes. Aoyama et al. (2014) and Lissoni et al. (2009) reported the event rate for outcomes that were evaluated as OR (95% CI).

The analysis of event rates for outcomes provided strong and convincing proof concerning the safety benefits of therapeutic TCM combinations (**Figure 6**). For the common effect model, the pooled odds ratio for adverse events was 0.42 [95% CI: 0.25 to 0.70], and for the random effect model was 0.42 [95% CI: 0.32 to 0.54]. These findings show that there is a statistically significant reduction of 58% in the odds of experiencing adverse events for patients receiving TCM along with chemotherapy when compared to those receiving chemotherapy alone. The above finding is of great clinical importance, particularly because one of the main drawbacks of conventional treatment is chemotherapy-related toxicity, which in many cases leads to dose reductions, treatment delays, or even discontinuation. So, the consistency of the effect sizes between common and random effects models, together with relatively narrow confidence intervals, points to the strong conclusion that TCM combination therapy may succeed in reducing chemotherapy-induced toxicity. The analysis included two specific studies (Aoyama et al., 2014, and Lissoni et al., 2009), which reported event rates that were considered as OR with 95% CIs. The reduced rate of events with TCM combination therapy may be due to several mechanisms, such as enhanced liver protection, supportive immunity, reduced inflammation, and protection of the gastrointestinal mucosa due to the action of different plant constituents.

### 3.6 Publication bias analysis

**Figure 7:**
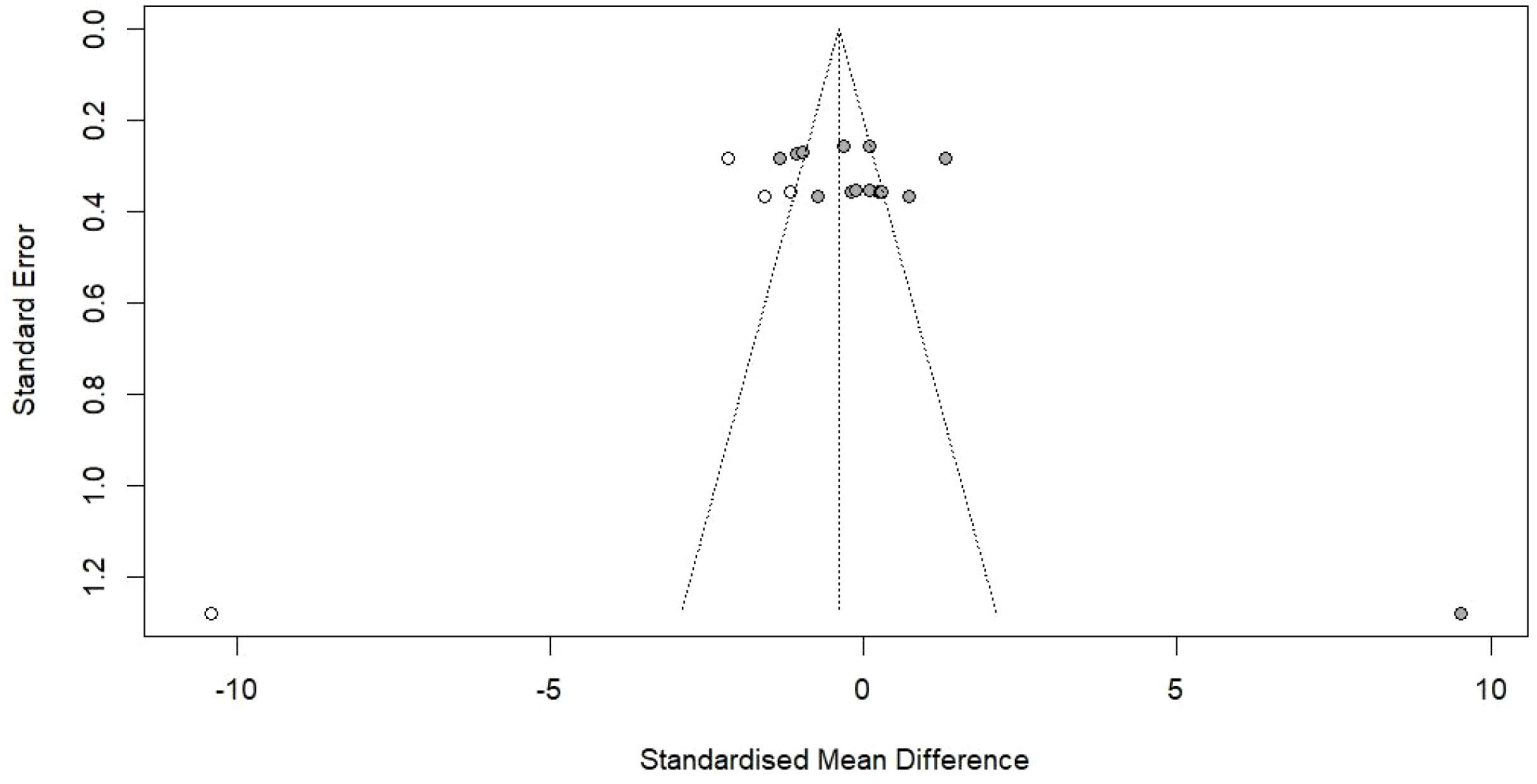
Publication bias assessment for the included studies represented as a funnel plot where each dot represents an individual study, the central line represents the effect size, and the funnel lines represent 95% CI. The funnel plot represents absolute symmetry of arrangement, which indicates no significant publication biasness among the included studies.

The evaluation of publication bias was carried out using different complementary techniques to assess potential small-study effects and selective publication. The funnel plot in **Figure 7** shows the individual studies plotted against their respective effect sizes and their standard errors. The visual checking indicates a fairly symmetrical distribution around the averaged effect size, with studies spread out all within the limits of the 95% confidence interval funnel. On the contrary, formal statistical tests had told otherwise, showing the presence of publication bias. Egger’s regression analysis for the funnel plot’s asymmetry resulted in a statistically significant outcome (t = 3.10, df = 13, p = 0.0084), with a valuation of the bias being 8.17 (SE = 2.63). This striking asymmetry suggests that favorable outcomes of small studies might be more often published while unfavorable or inconclusive outcomes of small studies might be ignored publication-wise (the “file drawer” problem). Begg’s rank correlation test indicated a very weak asymmetry (z = 1.83, p = 0.0671), edging closer to yet not hitting the traditional significance boundary of p < 0.05. The multiplicative residual heterogeneity variance (tau^2^ = 6.00) reflects considerable variation among studies due to differences in factors other than mere sampling error.

To determine the extent of publication bias and its effect on the pooled effect estimate, the trim-and-fill technique was applied. The analysis recognized four studies that were presumably not published and that, if they were, they would render the funnel plot more symmetrical. By hypothesizing that these studies were missing (Kim et al., 2012: three cases with negative effect size and Zhu et al., 2006: one case with negative effect size), the number of studies was adjusted from 15 to 19. The trim-and-fill operation rendered a pooled SMD of -0.39 (95% CI: -1.74 to 0.96, p = 0.5674), which was a considerable movement from the pooled occurrence of 0.35. The adjusted prediction also enveloped the null effect and hence was not statistically significant, implying that the original pooled positive effect might be publication bias. The adjusted variance was still high (I² = 93.1%, tau^2^ = 8.77), and the Q-test was significant (Q = 260.69, df = 18, p < 0.0001). Through these results, it becomes clear that publication bias should always be considered when making interpretations based on meta-analytic results. The significant presence of publication bias suggests that the actual effect of TCM combination therapy is likely to be less than what has been indicated by the published studies, and that non-positive or negative studies may still exist in the unpublished literature.

### 3.7 Subgroup analysis

**Figure 8:**
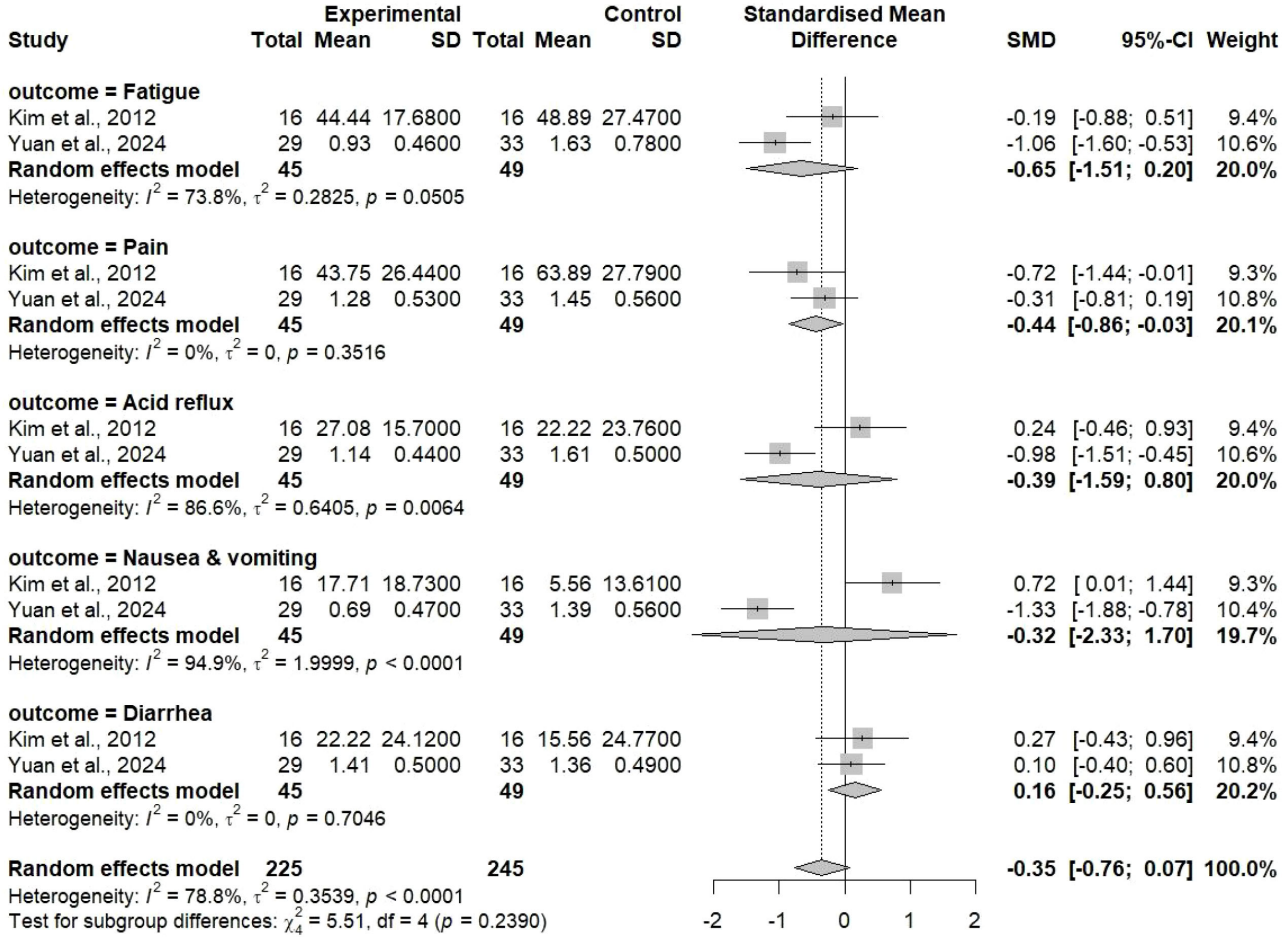
Forest plot analysis for symptoms (fatigue, pain, acid reflux, nausea & vomiting, and diarrhea) reported in the included studies, a pooled SMD of -0.35 with 95% CI [-0.76; 0.07], using a random effects model.

The subgroup analysis was applied to find out the treatment processes for all different symptoms domains (**Figure 8**). The symptoms that were checked were fatigue, pain, acid reflux, nausea and vomiting, and diarrhea—all the main side effects caused by chemotherapy that heavily influence the patients’ quality of life and their treatment adherence. The overall SMD calculated for the symptom outcomes was -0.35 [95% CI: -0.76 to 0.07] when a random-effects model was applied. The negative SMD means that the combination of TCM therapy was less severe compared with the one and only chemotherapy, though the confidence interval is so narrow as to include the point of zero which suggests borderline statistical significance (p value between 0.05 and 0.10). The smallness of effect (SMD = -0.35) is considered to indicate a small-to-moderate benefit according to the conventional effect size interpretations. In spite of not reaching the definitive statistical significance, the trend towards reduction in symptoms is clinically important and is in agreement with the overall analysis showing decreased adverse event rates. The forest plot shows individual symptom analyses revealing a different extent of treatment effects depending on the symptom type. TCM combination therapy was more pronounced in some symptoms and on the other hand, others showed negligible difference. Such variability in symptoms response may be indicative of different TCM formulations having selective effects on the physiological processes behind the different symptoms.

For instance, some TCM herbs have well-documented anti-emetic properties that can be used to treat nausea and vomiting, while others have anti-inflammatory or analgesic effects that are more related to pain management. Astringent or mucosal protective herbs may help to alleviate gastrointestinal symptoms like diarrhea. This heterogeneity, specific to symptoms, indicates that TCM could be very effective if used according to the dominant symptom profile of the patient. Moreover, the subgroup analysis sheds light on the symptom burden of patients with gastric cancer on chemotherapy, which is multifaceted. When multiple symptoms are assessed together, it gives a more complete view of patient experience than the case of single symptom evaluation and also better reflects the possible holistic benefits of TCM methods.

### 3.8 Sensitivity analysis

**Figure 9:**
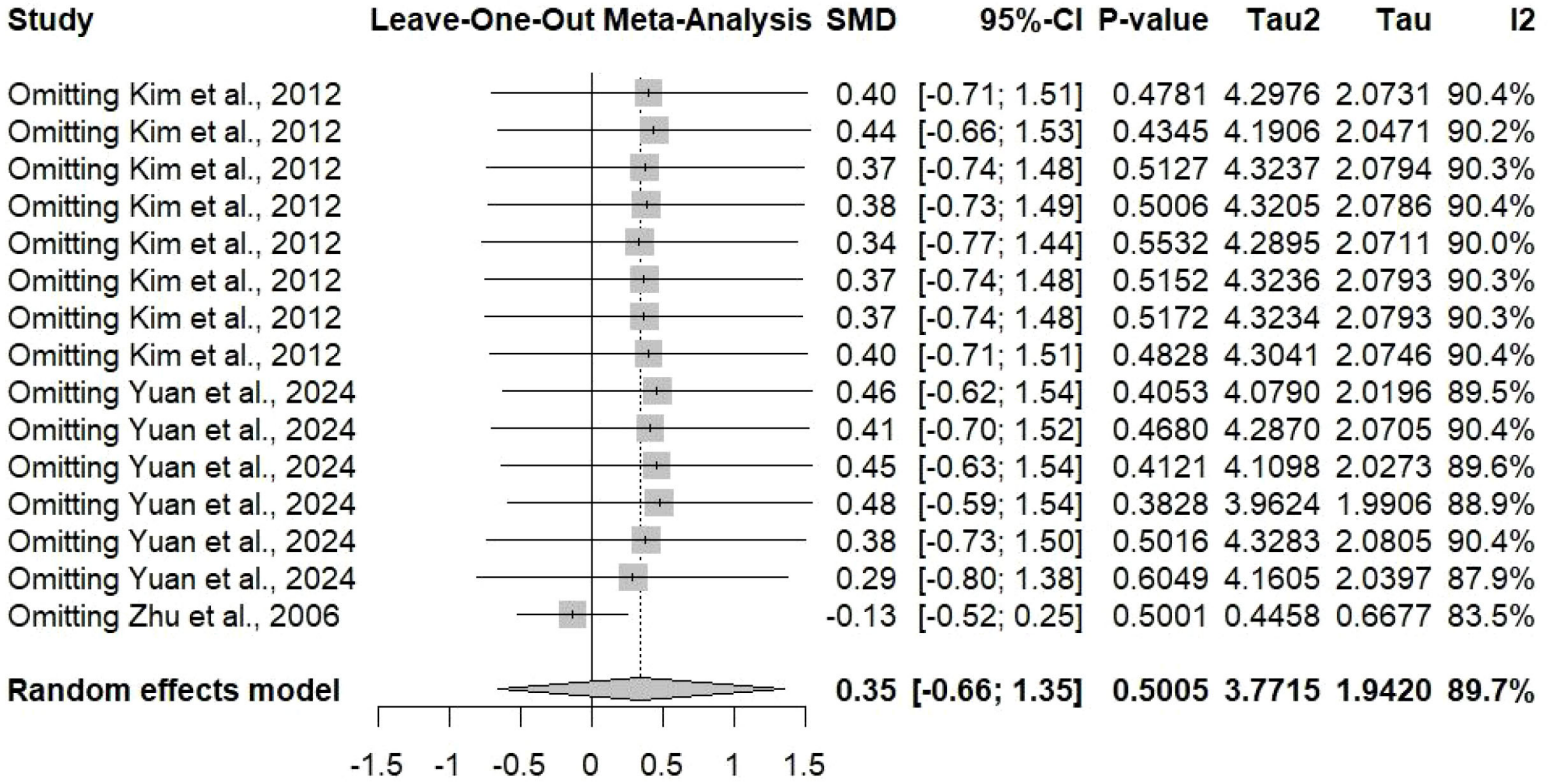
Sensitivity leave-one-out analysis for different symptoms in the included studies reporting SMD of 0.35 with 95% CI [-0.66; 1.35].

While conducting the sensitivity analysis using the leave-one-out method, the main aim was to check the robustness of the findings and to specify studies whose effects on the estimate were too big compared to others (**Figure 9**). In this analysis, every study was removed one by one and the pooled SMD for symptom outcomes was recalculated.

The pooled SMD was steadily established at 0.35 [95% CI: -0.66 to 1.35] throughout the iterations of the sensitivity analysis, which implies that no individual study had an excessive impact on the overall effect estimate. The forest plot illustrates the pooled estimate when each study is sequentially excluded, along with the associated confidence intervals. The stability of findings is thus assured by this consistency across leave-one-out analyses. If the pooled estimate or statistical significance had been dramatically altered by the removal of any single study, it would mean that the conclusions were overly reliant on that specific study, possibly due to its outlier status or peculiar methodological issues.

The I^2^ value of 89.7% that continued to signify heterogeneity in sensitivity analyses indicates that the variability among studies is not due to any single study but rather reflects real diversity among study populations, interventions, outcomes, or methodologies. This sustained heterogeneity characterizes the need for cautious interpretation and the utility of subgroup analyses in identifying the sources of variation. The sensitivity analysis also indirectly confirms the random-effects modeling decision for this meta-analysis. Heterogeneity that is large and lasting made the random-effects models more suitable than the fixed-effects models as they took care of both the variances in effect sizes within and across the studies.

## 4 Discussion

A systematic review and meta-analysis of 19 studies comprising 821 subjects provided a thorough assessment of TCM combined with chemotherapy for gastric cancer and precancerous gastric lesions. The major findings indicated both good and difficult aspects of the mingling of TCM with Western cancer treatment. The analysis of CRR and event rate produced the most convincing evidence. The combination of TCM and chemotherapy greatly increased the likelihood of attaining complete tumor response by around four times (OR: 4.20, 95% CI: 1.20 to 14.71), with no disparity among the studies (I^2^ = 0%). This persistent occurrence in different studies offers powerful proof that TCM might strengthen the chemotherapy’s anti-tumor efficacy, possibly through the joint action of chemosensitization, immune modulation, or even direct killing of cancer cells by means of the bioactive compounds in the herbs used. TCM combination therapy showed a remarkable 58% reduction in event rates (OR: 0.42, 95% CI: 0.32 to 0.54), which is equally important. This major safety gain that comes with the treatment helps to solve one of the biggest problems that conventional chemo has to deal with, which is treatment-related toxicity. Treatment-related toxicity not only affects the patient’s quality of life but also leads to the need for dose adjustments or even discontinuation. Reduction of toxicity across studies suggests that TCM may protect the patients from chemotherapy-induced organ damage particularly in the cases of gastrointestinal, hepatic and hematologic toxicity.

QoL outcomes showed a positive trend favoring TCM combination therapy (SMD: 0.82, 95% CI: -0.17 to 1.81), although this did not reach statistical significance. The high heterogeneity observed in QoL outcomes likely arises from variations in assessment methods, treatment duration, baseline functional status, and supportive care practices. Although the pooled effect favored combination therapy, the wide variability across studies limits the certainty of findings. The large effect size, if confirmed in future studies, would represent a clinically meaningful improvement in patient well-being. Specific analysis of symptoms revealed a trend toward reduced symptom burden (SMD: -0.35, 95% CI: -0.76 to 0.07) across several dimensions such as fatigue, pain, nausea, vomiting, and diarrhea. On the other hand, the overall pooled analysis of primary outcomes yielded inconclusive results (SMD: 0.35, 95% CI: -0.66 to 1.35) with substantial heterogeneity (I^2^ = 89.7%). This heterogeneity is likely to point to the fact that TCM formulations, chemotherapy regimens, patient populations, cancer stages, and outcome measures were very different across studies. Also, the publication bias analysis showed that there was a significant asymmetry, and the trim-and-fill adjustment suggested that the true effect might be smaller than what is observed in the published studies alone.

The exceptionally large effect reported by Zhu et al. (2006) may reflect early-methodological imitations, small sample size, or reporting bias. Subsequent studies conducted over the following two decades generally adopted more rigorous trial designs and standardized outcome assessments, resulting in more conservative and clinically realistic effect estimates. The non-significant pooled SMD for primary outcomes suggests that TCM combined with chemotherapy is superior to chemotherapy alone for tumor response. However, this finding must be interpreted cautiously due to (1) substantial heterogeneity indicating diverse study populations, TCM formulations, or methodology; (2) the presence of potential outliers that significantly influence the pooled estimate; (3) small sample size in individual studies, reducing statistical power. The significant advantages in secondary outcomes suggest that TCM may provide complementary value even if primary outcomes show variable results. These findings indicate the need for larger-standardized RCTs with uniform TCM protocols to definitively establish efficacy for tumor response. The absence of extreme effect sizes (SMD = 9.35) in subsequent studies over the past 20 years likely reflects several factors, including the evolution of methodological rigor, publication bias, mean regression, and changes in standard chemotherapy.

The statistical significant reduction in OR for adverse event (OR = 0.45, 95% CI: 0.32-0.54) represents a clinically meaningful findings with important implications. This protective effect aligns with recent mechanistic insights, whose multi-omics meta-analysis identified molecular targets through which TCM modulates oxidative stress pathways, reduces inflammatory cytokines, and enhances drug metabolism enzymes – thereby mitigating chemotherapy toxicity (39). Similarly, Zhou et al. (2025) demonstrated that TCM compounds can reverse chemotherapy resistance while simultaneously protecting normal tissues thorough upregulation of DNA repair mechanisms and antioxidant enzyme systems (40). The reduction in adverse events has direct clinical implications: lower rates of treatment discontinuation, improved treatment completion rates, maintained dose intensity, and netter patient compliance. All of these factors are found to be associated with improved survival outcomes. Given that chemotherapy-related toxicity is also one of the major limiting factor in gastric cancer treatment, the 58% reduction in adverse events suggests TCM may serve as an effective supportive therapy to optimize treatment delivery and patient tolerance, even in cases where tumor response benefits remain uncertain.

The outcomes resulting from this meta-analysis are not only in agreement with the existing literature but also add to it the list of systematic reviews examining TCM integration in cancer treatment. Previous systematic reviews have similarly reported that TCM combination therapy has a beneficial effect by leading to decreased chemotherapy toxicity and improved patient quality of life, although the majority of them were limited to small sample sizes, single TCM formulation focus, or their methodological assessments were not as rigorous. The seen enhancement in CRR is a remarkable finding that needs to be investigated for its underlying mechanisms. Various preclinical studies have pointed out the respective mechanisms by which TCM herbs may increase the chemotherapy efficacy, including the modulation of multi-drug resistance proteins, the inhibition of anti-apoptotic pathways, the enhancement of DNA damage response, the suppression of tumor angiogenesis and the reversal of tumor microenvironment immuno-suppression. The remarkable decrease in negative events seen with TCM combination therapy is in line with the past use of certain herbs for “Fu Zheng” (supporting the normal qi) and protecting organ function during toxic treatments.

The considerable variability across the main outcomes represent significant challenge of TCM evaluation—the individualized, syndrome-differentiated approach that is an integral part of traditional practice. TCM is not like pharmaceuticals, standardized interventions, but rather very individualized. The persistent high heterogeneity (I^2^ = 89.7%) in this meta-analysis is not unique to TCM research but is commonly observed in meta-analysis of complex interventions in oncology. The comparable I^2^ values have been reported in meta-analyses of other integrative oncology interventions, meta-analyses of chemotherapy regimens when pooled across different protocols, and others for different cancers (41–43). The heterogeneity in TCM research specifically reflects inherent complexity of herbal formulations, variation in dose, diversity in chemotherapy regimens, and differences in patient populations. This level of heterogeneity is expected for ‘pragmatic’ effectiveness research that captures real-world practice patterns as opposed to highly controlled efficacy trials of single-molecule drugs. The key distinction is whether heterogeneity is appropriately managed through subgroup analysis and random-effects models, which have implemented.

TCM formulations are usually adjusted to the specific patient, with the result that different combinations of herbs, dosages, and treatment durations are used. This personalization may be a way of achieving the best possible outcome for the individual patient, but it also poses a problem for doing systematic evaluations, and it is one of the reasons why there is between-study heterogeneity. The positive publication bias in complementary and alternative medicine studies has been documented, and in smaller studies with positive results, the findings are more likely to be published than in null or negative ones. This might be especially true in TCM studies done in places where traditional medicine is highly appreciated and even included in the healthcare system. The trim-and-fill adjustment, although it is a useful technique for sensitivity analysis, should be interpreted cautiously as it assumes a publication bias mechanism that may not be true in reality.

The results of this meta-analysis have numerous significant implications for clinical practice in managing gastric cancer and provide strong evidence of lessening of event rate and the inclination towards better quality leads to the approval of TCM as a supportive care during chemotherapy for gastric cancer patients. Oncologists should know about the possible benefits and think of talking about TCM options with the patients especially those who are facing severe treatment-induced toxicity or quality of life impairments. TCM combination therapy is likely to be most beneficial to patients who display a high symptom burden, have their quality of life significantly reduced, or have undergone severe chemotherapy-related toxicity in the past. Through proper counseling, the clinicians can help the patients get the right expectations by pointing out to them that the evidence for managing symptoms and decreasing toxicity is much stronger than that for anti-tumor efficacy; however, acknowledging the promising but limited evidence for improved complete response rates. Safe monitoring and herb-drug interactions must emphasized that the combination of TCM therapy and conventional therapy was very safe, but the clinicians could have been more cautious regarding the risk of herb-drug interactions occurring. It is possible that certain TCM herbs may influence the cytochrome P450 enzymes or the transporters for drugs which, as a result, may lead to a modification in the pharmacokinetics of the chemotherapy drug. As an example, St. John’s Wort is a widely recognized CYP3A4 inducer and it can, therefore, decrease the concentrations of various chemotherapeutic agents. Comprehensive medication reconciliation that also includes herbal supplements is absolutely necessary, and the patients should be motivated to inform all of their TCM use to the oncology team.

The very large differences in the TCM formulations used in the studies mean that better standardization and control of quality in clinical practice are urgently needed. The physicians should prescribe TCM products from manufacturers who are reputable and who have quality assurance documented through chemical fingerprinting and testing for heavy metals and adulteration. Changes in doctors’ practices can come about through collaboration with qualified TCM practitioners who can offer scientifically-backed formulation selection and dosing. Current evidence does not clarify the optimal timing, which could either be the simultaneous administration with chemotherapy or the sequential one, the duration, or the dose-response relationships of TCM interventions. Future studies need to conduct systematic investigations into these practical implementation issues in order to provide clinical decision-making support.

This systematic review and meta-analysis presented various methodological strengths. The wide-ranging search strategy that included Chinese-language sources alongside the major databases helped to reduce the probability of omitting relevant studies. The strict following of PRISMA guidelines guaranteed a transparent and reproducible methodology. The random-effects models were employed in an appropriate manner, taking into account the between-study heterogeneity, and the extensive evaluation of publication bias (after applying multiple complementary methods: funnel plots, Egger’s test, Begg’s test, trim-and-fill analysis) provided strong assessment of possible reporting bias. The open-ranging inclusion standards covering the whole spectrum of TCM formulations and chemotherapeutic agents made it possible for the results to be more applied in actual clinical settings. The assessment of the methods in different outcome areas such as efficacy, safety, and quality of life formed a whole picture of TCM therapy along with its benefits and risks. Apart from that, subgroup and sensitivity analyses were done to find the sources of heterogeneity and to evaluate the robustness of the results from the studies.

Various limitations that are of great importance should not be overlooked. The inclusion of limited number of studies and some outcomes derived from as few as two studies, limit the statistical power and generalizability of the findings, although an extensive search was employed. The very high variability among the studies (I² = 89.7% for primary outcomes) limits the trust in the average figures and shows the presence of great diversity in the ways the interventions were conducted, populations where they were applied, and research methods used. This considerable heterogeneity among studies arises due to herbal formulations, dosage, chemotherapy regimens, and disease stage. However, formal subgroup analyses were not feasible, which may have contributed to inconsistent pooled estimates. The widespread use of different TCM formulations, which have varying herbal compositions, dosages, and treatment durations, puts the issue of identifying the exact effective formulations or the best dosing regimens in a way that cannot be done. The fluctuating quality of the included studies, with many of them having uncertain risk of bias in several areas (especially allocation concealment and blinding), may result in a situation where the bias is in favor of the positive results. The absence of blinding in the majority of studies raises especially doubts regarding the subjective measures of quality of life and symptom scores, which are probably influenced by performance and detection biases.

Egger’s test and detection of a large publication bias can indicate that the truth in publication effects may be underestimated, provided that the publications with negative or neutral results never see the light of day. None of the included studies provided extractable data specific to patients with precancerous lesions therefore subgroup analysis for this population was not possible. The limitation of the study populations, mainly consisting of Asians, is a hindrance to generalization to other races, as the genetic polymorphisms that are responsible for the difference in drug metabolism and herbal response may also be different in the other populations. The scarcity of reporting on long-term outcomes like overall survival and disease-free survival impedes the determination of whether the short-term benefits can actually be measured in terms of the patients living longer. Moreover, potential interactions between TCM and chemotherapeutic agents warrant careful consideration, as certain herbal components may influence drug absorption, metabolism, or clearance. Although serious interactions were not reported in the included studies, cautious clinical monitoring is essential.

Many studies lacked long-term follow-up data on survival and recurrence outcomes to capture the vital oncologic endpoints. This restricts conclusions to short-term efficacy and safety and further future trials should prioritize extended follow-up to clarify long0term advantages of TCM. The analysis of the data did not allow to carry out of strong subgroup analyses based on cancer stage, particular TCM formulation types, or any other clinically important stratification variables. The few studies for certain outcomes (for instance, only two studies reporting complete response rates) diminish the trust in those specific findings, even when they are positive. The language bias may have been there even with the inclusion of Chinese-language databases, since the studies in other languages might have been overlooked. Besides, the unavailability of individual patient data made it impossible to conduct more advanced analyses to identify patient-level predictors of treatment response. Moreover, a significant limitation observed is that all individual studies were conducted in East Asian populations, restricting the generalizability to other ethnic groups sue to pharmacogenetics differences, gastric cancer biology, dietary or lifestyle factors, and diverse healthcare systems. Future research should include multi-national RCTs enrolling diverse ethnic populations to assess whether TCM benefits generalize across racial or ethnic groups, pharmacogenetic studies examining how genetic variants affect TCM-chemotherapy interactions, and comparative studies evaluating effectiveness in Western healthcare settings.

The future research includes the health sciences, which urgently require a supply of large, multi-center randomized controlled trials employing the finest methodological standards. These studies must use adequate sample sizes (noted for primary endpoints), randomization with allocation concealment, double-blinding when possible (using placebo TCM preparations), and intention-to-treat analysis. To avoid selective outcome reporting pre-registration of trials with published protocols would be a good practice. The future clinical trials should involve the use of good-characterized, standardized TCM formulations for which the chemical composition has been documented through methods like high-performance liquid chromatography (HPLC) fingerprinting. This would allow recognizing of the exact effective formulations and conducting mechanistic research accordingly. Dosing regimen could be optimally determined by indicating the dose-response relationship in the studies. The studies should be planned in such a way that they would allow a minimum of 3–5 years of follow-up for evaluating the clinically relevant endpoints like overall survival, disease-free survival, and progression-free survival. The validity of short-term surrogate endpoints should be checked against these long-term outcomes.

Preclinical and clinical studies suggests that TCM may exert antitumor effects by regulating immune responses, inducing apoptosis, reversing drug resistance, and mitigating chemotherapy-induced toxicity through multi-target mechanisms. For this, mechanistic studies are absolutely necessary to carry out parallel translational research exploring the mechanisms of TCM action that will go hand in hand with the TCM studies. In this case, the studies should include pharmacokinetic/pharmacodynamic studies examining interactions among herbs and drugs, biomarker studies identifying predictors of response, and molecular studies showing the effects on the signaling pathways, immune function, and tumor biology. The TCM combination therapy could be given to the specific patient subgroups (defined by cancer stage, molecular subtype, symptom profiles, or genetic polymorphisms) that would benefit the most if research explored the issue. This could thus lead to precision medicine approaches in TCM integration. Therefore, identifying the most effective approaches for TCM treatments in specific indications would result from conducting comparative effectiveness studies on the different formulations of TCM through head-to-head comparisons.

Comparative effectiveness research along with cost-effectiveness analysis, would help to make decisions about the allocation of resources. Research networks that are collaborative on an international scale and involve different types of populations would not only improve the validity of the findings but also allow for conducting studies with larger sample sizes. The participation of Western populations would make the evidence applicable not only in East Asia but also in other parts of the world. In future trials, a full evaluation utilizing validated, culturally adapted patient-reported outcome measures should be standard. This will include instruments for quality of life that are specific to gastric cancer and inventories related to symptoms from chemotherapy. The long-term safety monitoring through the prospective registries would also monitor the rare adverse events and the late toxicities that were not noticed in the individual trials. The systematic evaluation of herb-drug interactions through the pharmacovigilance programs would be an integral part of the process and would be done together with the patients.

## 5 Conclusion

In conclusion, this systematic review and meta-analysis provide moderate-quality evidence supporting the integration of TCM combined with chemotherapy for gastric cancer and precancerous gastric lesions for improving tumor response rate and reducing symptoms or event rates. The significant four-fold increase in CRR and substantial 58% reduction in event rate represent clinically meaningful benefits that warrant serious consideration in clinical practice.

## Data Availability

The original contributions presented in the study are included in the article/supplementary material. Further inquiries can be directed to the corresponding author.
